# Tuberculous colon perforation mimicking acute appendicitis: A case report and review of the literature

**DOI:** 10.1002/ccr3.1795

**Published:** 2018-09-28

**Authors:** El‐Rifai Nahida, Harissi Hassan, Daoud Nabil, Leteurtre Emmanuelle, Gottrand Frédéric

**Affiliations:** ^1^ Department of Pediatrics Makassed General Hospital Beirut Lebanon; ^2^ Department of Surgery Makassed General Hospital Beirut Lebanon; ^3^ UMR‐S 1172 ‐ JPARC ‐ Jean‐Pierre Aubert Research Center Inserm, CHU Lille Université Lille Lille France; ^4^ Division of Gastroenterology, Hepatology and Nutrition Jeanne de Flandre Children Hospital CHU‐Lille Univ. Lille Lille France

**Keywords:** abdominal pain, acute appendicitis, intestinal tuberculosis

## Abstract

Acute appendicitis is the most common surgical condition encountered in the pediatric age group but the clinical presentation could be misleading. This highlights the importance of exploratory laparotomy even in the absence of systemic manifestations in order not to miss a serious underlying pathology such as intestinal tuberculosis.

## 1 INTRODUCTION

Abdominal tuberculosis (TB) is a rare manifestation of extrapulmonary TB with a prevalence ranging from 1% to 5% of the pediatric cases of pulmonary TB. It may involve the gastrointestinal tract, peritoneum, mesenteric lymph nodes, and genitourinary tract. Early diagnosis of intestinal TB remains difficult due to its nonspecific clinical presentation and its high resemblance to malignancy or inflammatory bowel diseases.^1^ An acute abdominal presentation is relatively nonspecific and a diagnosis of abdominal TB can be easily missed. We present here a patient, who presented with acute onset right lower quadrant pain suggestive of acute appendicitis. Later on, the diagnosis of abdominal TB was confirmed after histopathological examination of mesenteric lymph nodes.

## 2 CASE REPORT

A 10‐year‐old male patient, previously healthy, attended our Emergency Department complaining of abdominal pain of 12 hours duration. The pain was periumbilical, moderate in intensity, and associated with nausea. The pain became more severe and shifted to the right lower quadrant. There were no other associated symptoms. The past medical history was irrelevant. There was no reported fever neither weight loss nor night sweats. He was not BCG‐vaccinated. Upon presentation, the patient was conscious, oriented, in pain, and afebrile. Vital signs were within normal range. The abdomen was soft, tender on deep palpation of the right lower quadrant. The McBurney sign was positive. The rest of physical examination was unremarkable. Laboratory investigations revealed a hemoglobin of 11.2 g/dL (range 11‐13.3 g/dL), hematocrit 36.5 (range 31.5‐38 g/dL), WBC 11 000/mm^3^ (neutrophils 82%, lymphocytes 11%) [range 5700‐9900/mm^3^], platelets 381 000/mm^3^ (range 227 000‐350 000/mm^3^), and MCV of 71 fL/red cell (range 78.2‐83.9 fL/red cell). The C‐Reactive Protein (CRP) was 2 mg/dL (normal <0.3 mg/dL). Since these symptoms were consistent with appendicitis, the patient was taken for emergency surgery without abdominal imaging. During surgery, there was minimal appendicular inflammation with pus in the right lower quadrant. Further exploration revealed a mass in the mesentery. There was a sealed perforation between the right colon and the small intestine without spillage, causing large mesenteric lymph nodes. The colon was released, edges debrided, and the perforation was closed. Biopsies taken from the colon and lymph nodes were sent for pathology. Patient was started on ampicillin, gentamycin, and ceftriaxone. The postop period was uneventful. A culture taken from the pus did not grow any organism. Antibiotics were continued for 7 days. Histopathological examination of the colon biopsies revealed granulomatous inflammation with multinucleated giant cells with caseating granuloma. The Ziehl‐Neelsen stain was negative. The tuberculin skin test (TST) for the patient examined after 48 hours was positive with an induration of 25 mm in diameter. The interferon gamma release assays (IGRAs) was positive. So the diagnosis of tuberculosis was confirmed. Chest X‐ray and CT scan of the chest were normal. PCR for tuberculosis on abdominal fluid was negative. The patient was started on quadruple therapy (isoniazid, ethambutol, pyrazinamide, and rifampin), and the patient was discharged home.

## 3 DISCUSSION

Acute abdominal pain is one of the most common complaints in children presenting to the emergency department with acute appendicitis being the most common surgical cause. However, acute abdomen pain may not be easily diagnosed in young children based on the clinical presentation because of their poor ability to express themselves. In addition, many diseases can mimic acute appendicitis ranging from simple pinworm infestation to more serious pathologies such as non‐Hodgkin's lymphoma.^2^, ^3^

Our patient presented with acute onset abdominal pain suggestive of acute appendicitis. However, the clinical presentation was misleading since the final diagnosis was abdominal TB. Fever, abdominal pain, abdominal distension, ascites, and weight loss are the most common presenting symptoms of abdominal TB reported in the literature.^4^

Surprisingly, in our case, the patient presented with acute onset abdominal pain in the absence of long‐lasting symptoms which make the diagnosis of abdominal TB even more unlikely. Later on, the diagnosis was suspected in the presence of a mass in the mesentery with sealed colon perforation into the small intestine. One should eliminate in this case the possibility of Crohn's disease in which localized peritonitis could be the first manifestation of the disease in 1%‐2% in Western countries and the misdiagnosis rate between CD and ITB is 50%‐70%.

Furthermore, according to Bass et al, preoperative findings of a low hemoglobin level (10.4 + 1.0 vs 13.3 + 0.2) and mean corpuscular volume (MCV) (72.5 + 3.4 vs 84.1 + 0.5) values, and higher platelets values (444.8 + 42.2 vs 275.6 + 8.0) in a child presenting with appendicitis warrant further evaluation for CD, as prompt diagnosis allows for optimal treatment and quality of life for these patients.^5^

Our patient was having a low hemoglobin level (11.2 g/dL) with a high platelets counts 381 000/mm³ and an MCV of 71 fl/red cell which raised the possibility of Crohn's disease.

Recently, abdominal imaging in acute appendicitis resulted in improved diagnostic accuracy and reduction of negative surgeries. Performing a CT scan in our patient would have intestinal TB suspected and the management changed but the clinical picture was highly suggestive of acute appendicitis. This is the reason why the patient was operated without further imaging.

The diagnosis of intestinal TB (ITB) could be established when at least one of the following criteria is met: (a) histological demonstration of caseating granuloma, (b) identification of acid‐fast bacilli (AFB) in a histological specimen, (c) clinical, colonoscopic, radiological and/or surgical evidence of ITB associated with proven TB elsewhere and dramatic response to anti‐tuberculous treatment.

In our case, tuberculosis was suspected initially based on histopathology and was confirmed by the interferon gamma release assays (IGRAs). A recent meta‐analysis of the most important studies indicates that, although not sensitive enough, IGRAs provide good specificity for the accurate diagnosis of intestinal TB.

Quadruple therapy remains the most effective regimen of intestinal TB with the combination of isoniazid, rifampicin, pyrazinamide, and ethambutol for 2 months then isoniazid and rifampicin for 8 months. The patient was started on quadruple therapy, and he responded well.

## 4 CONCLUSION

Although acute appendicitis is the most common surgical condition encountered in children, the clinical presentation of the patient could be misleading. Our case highlights the importance of exploratory laparotomy in patients with acute appendicitis even in the absence of systemic manifestations in order not to miss a serious underlying pathology such as intestinal tuberculosis.

## CONFLICT OF INTEREST

None declared.

## AUTHOR CONTRIBUTIONS

ERN, HH, and GF: contributed to the writing and discussion of the case. ND: underwent the surgery for the patient. LE: contributed to the histopathological examination of the biopsies taken during surgery and contributed to the discussion of the case.
